# The Prevalence of Autoantibodies in Complex Regional Pain Syndrome Type I

**DOI:** 10.1155/2015/718201

**Published:** 2015-02-08

**Authors:** Maaike Dirckx, Marco W. J. Schreurs, Marissa de Mos, Dirk L. Stronks, Frank J. P. M. Huygen

**Affiliations:** ^1^Center for Pain Medicine, Erasmus MC, University Medical Center, Postbus 2040, 3000 CA Rotterdam, Netherlands; ^2^Department of Immunology, Erasmus MC, University Medical Center, Postbus 2040, 3000 CA Rotterdam, Netherlands

## Abstract

Autoimmunity has been suggested as one of the pathophysiologic mechanisms that may underlie complex regional pain syndrome (CRPS). Screening for antinuclear antibodies (ANA) is one of the diagnostic tests, which is usually performed if a person is suspected to have a systemic autoimmune disease. Antineuronal antibodies are autoantibodies directed against antigens in the central and/or peripheral nervous system. The aim of this study was to compare the prevalence of these antibodies in CRPS patients with the normal values of those antibodies in the healthy population. Twenty seven (33%) of the 82 CRPS patients of whom serum was available showed a positive ANA test. This prevalence is significantly higher than in the general population. Six patients (7.3%) showed a positive result for typical antineuronal antibodies. This proportion, however, does not deviate from that in the general population. Our findings suggest that autoantibodies may be associated with the pathophysiology of CRPS, at least in a subset of patients. Further research is needed into defining this subset and into the role of autoantibodies in the pathogenesis of CRPS.

## 1. Introduction

Complex regional pain syndrome (CRPS) is a collection of locally appearing painful conditions following trauma which chiefly occur distally and exceed in intensity and duration the expected clinical course of the original trauma.

The pathophysiology is complex and still not completely understood. It is reasonable to assume that different mechanisms, for example, inflammation, hypoxia, central sensitisation, and neuroplasticity, are involved in a complex network of interactions, resulting in a broad range of signs and symptoms [1].

The involvement of the immune system in the pathophysiology of CRPS is appreciated for several reasons. First, CRPS shows several clinical characteristics of an inflammatory disease, including pain, redness, swelling, and warmth [2]. Additionally, levels of proinflammatory cytokines are elevated in blister fluid from CRPS affected limbs [3, 4]. CRPS shows a beneficial response to treatment with inhibitors of inflammation, such as corticosteroids [5].

Complementary is the fact that, similar to many other chronic inflammatory diseases, CRPS displays a female predominance [6] and associations with distinct HLA alleles [7–9]. The incidence of CRPS is higher in patients with chronic inflammatory disorders, such as asthma [10] and multiple sclerosis [11].

Autoimmunity has been suggested as one of the underlying mechanisms in the pathophysiology of CRPS. There are several arguments that point in this direction. First, IgA-antibodies to campylobacter were present in CRPS patients with short disease duration [12] and an increased seroprevalence of Parvovirus B19 in CRPS patients compared to controls has been reported [13, 14]. Both infectious agents have previously been implicated in the induction of autoimmune diseases.

Second, immunohistochemistry has revealed the presence of autoantibodies against nervous system structures in at least a part of the CRPS patients, included in a study by Blaes et al. [15]. Another study showed that about 30–40% of CRPS patients have surface-binding autoantibodies against an inducible autonomic nervous system autoantigen [16]. Third, a subgroup of CRPS patients, that is, those who developed CRPS with only a minimal preceding trauma, showed a much stronger immune response against nervous tissue compared to the whole group [12]. Fourth, animal studies have demonstrated that the transfer of IgG antibodies from CRPS patients to mice causes abnormal behaviour and motor function in these mice [17]. And finally, treatment with intravenous immunoglobulin can reduce pain in refractory CRPS [18].

These results suggest that CRPS is associated with autoimmunity, including an autoantibody-mediated immune process, at least in a part of the patients. Interestingly, CRPS is even considered as prototype of a novel kind of autoimmune disease [19].

Autoimmune diseases are often associated with an increased prevalence of positive testing for antinuclear antibodies (ANA). These autoantibodies are reactive with antigens in the nucleoplasm. ANA are probably present in the circulation of all human beings, but the employed test is considered positive when titres are elevated significantly above the normal serum level [20]. Screening for ANA is one of the diagnostic tests which is usually performed if a person is suspected to have a systemic autoimmune disease [21].

Antineuronal antibodies, often called “onconeural antibodies” given their paraneoplastic nature in many cases, are autoantibodies directed against antigens in the central and/or peripheral nervous system. Antineuronal antibodies against intracellular antigens in general are not thought to be pathogenic. On the contrary, the antineuronal antibodies directed against cell surface antigens are themselves disease mediating. In contrast to what the name “onconeural” suggests, antineuronal antibodies are not strictly related to cancer [22].

The aim of the present study was to further explore CRPS as a potential autoantibody-associated autoimmune process. For this purpose, we compared the prevalence of CRPS patients with a positive test for antinuclear antibodies and for antineuronal antibodies with the prevalence in the healthy population.

## 2. Materials and Methods

This study was approved by the Medical Ethics Committee of the Erasmus MC Rotterdam (MEC-2012-037).

### 2.1. Patients

Our Department, a University Center for Pain Medicine, serves as an expert center for CRPS patients. Both acute and chronic CRPS patients visit the clinic on their own initiative or on referral by GP's or medical specialists. There is a weekly outpatient clinic especially for CRPS patients, led by physicians with clinical and research experience in CRPS.

All patients who visited the Center for Pain Medicine between 2001 and 2007 and fulfilled the Harden-Bruehl diagnostic criteria for CRPS [23] were invited to participate in ongoing CRPS studies.

For all patients signs (subjective) and symptoms (objective), that is, the presence or absence of allodynia, hyperalgesia, dystonia, bilateral difference in temperature, difference in colour, difference in sweating, difference in motor range, and difference in strength, were recorded.

In each patient who participated in a study, venous blood was drawn. Serum of this blood was stored with permission to use in future research. For the current study, serum of 82 patients was available. All these patients were classified as CRPS type 1. The characteristics of the 82 patients are described in Table 1.

### 2.2. Laboratory Tests

Venous blood samples were centrifuged immediately after collection at 3000 rpm during 10 minutes and serum was stored at minus 80 degrees Celsius until use.

Antinuclear antibodies (ANA) were determined according to international recommendations [24] with a commercially available test system, according to manufacturer's instructions. Briefly, HEp-2000 cells (Immuno Concepts, Sacramento, CA) were incubated with 1 : 80 diluted patient serum. After washing, bound autoantibody was detected using fluorescein (FITC-) conjugated anti-human IgG (Immuno Concepts) and visualized at 488 nm by fluorescence microscopy. ANA results were considered positive if at least weak positive nuclear staining of HEp-2000 cells was observed. Borderline results were discarded.

Antineuronal antibodies were determined according to international guidelines [25] with a commercially available test system, according to manufacturer's instructions.

Briefly, primate cerebellar cryosections (IMMCO Diagnostics, Buffalo, NY) were incubated with 1 : 400 diluted patient serum. After washing, bound autoantibody was detected using fluorescein (FITC-) conjugated anti-human IgG (IMMCO diagnostics) and visualized at 488 nm by fluorescence microscopy. Results were considered positive if at least weak positive staining of neuronal nuclei (antineuronal nuclear antibody, ANNA) or Purkinje cells (Purkinje cell cytoplasm antibody, PCA) was observed. Borderline results were discarded as well as false positive neuronal nuclear staining in the presence of a positive ANA.

Since literature reference varies regarding prevalence of autoantibodies in healthy individuals, mostly due to methodology, we did not use literature reference for comparison. Instead, the results of ANA and antineuronal antibody obtained in CRPS patients were compared to those we obtained ourselves in parallel using serum obtained from randomly selected healthy blood bank donors, using identical methodology as described above.

### 2.3. Statistical Analysis

Descriptive statistics were used to determine the (multiple response) frequencies of the demographic variables and the outcome parameters and to describe measures of central tendency and of variability, dependent on the shape of their distribution. Testing for normality of the distributions was performed using the Kolmogorov-Smirnov test. The difference between the proportion of CRPS patients with positive (nuclear or neuronal) antibodies and that in the healthy population was analyzed using the Fisher's Exact Test, 2-sided. The same test was applied to evaluate the difference in proportion of signs and symptoms between (1) the patients positive and those negative for antinuclear antibodies and (2) between the patients positive and those negative for antineuronal antibodies.

Difference in duration of the CRPS between patients with positive and those with negative antinuclear antibodies was tested using the Mann-Whitney* U* test.

Analyses were performed using IBM SPSS Statistics 21.

## 3. Results

Twenty seven (33%) of the 82 included CRPS patients showed a positive result for antinuclear antibodies. This proportion is significantly higher compared to that in the healthy population (*n* = 90), in which we observed 4% ANA positivity (*P* < 0.001).

The observed ANA immunofluorescence patterns were diverse, including homogeneous, speckled and nucleolar patterns. See Table 2.

No statistically significant difference was found in the proportion of patients with an ANA positivity between patients with a cold and those with a warm CRPS, respectively, 34.1% and 32.3% (*P* = 0.99). Likewise, no statistically significant difference in duration of the CRPS was found between the patients with a positive test for ANA and those with a negative test (*P* = 0.66).

Six (7.3%) of the 82 included CRPS patients showed a positive result for antineuronal antibodies. This percentage does not deviate from that in the healthy population (7.5%).

As indicated by the immunofluorescence pattern on the cerebellum substrate, the majority of reactivity was directed against neuronal nuclei (ANNA). In addition, reactivity against Purkinje cell cytoplasm (PCA) was observed. See Table 3. However, the immunofluorescence pattern in the healthy population indicated reactivity to basket neurons and/or neurofilaments, as opposed to the ANNA and PCA patterns observed in the CRPS patients.

Two patients showed both a positive ANA test (speckled pattern) and a positive result for antineuronal antibody (ANNA).

No statistically significant differences in signs or symptoms between patients positive and those negative for antinuclear antibodies were found. The same was applied to patients positive and those negative for antineuronal antibodies. For all proportional differences 0.13 = *P* ≤ 1.

## 4. Discussion

To gain more insight in the potential role of systemic and/or organ-specific autoimmunity in the pathophysiology of CRPS, we studied the prevalence of both antinuclear antibodies (ANA) and antineuronal antibodies in CRPS patients.

The reported prevalence of ANA in healthy individuals is up to 20% or more. The prevalence of ANA depends, however, on various factors including age, gender, and methodology [26]. Using our method of ANA testing, the prevalence in healthy individuals was 4%.

In our CRPS study sample we found a statistically significant higher positive ANA prevalence (33%) compared to that in the healthy population. To correct for a possible confounder age, since the prevalence of positive testing for ANA in the general population is higher amongst people aged above 65 years (up to 30%), we excluded the CRPS patients aged above 65 years (two patients). The positive ANA prevalence in CRPS remained significantly higher, 30%.

Diverse ANA patterns were observed in CRPS, including homogeneous, speckled, and nucleolar patterns. Either pattern can be observed in systemic autoimmune disease but is not specific to any particular autoimmune disease [27].

The prevalence of antineuronal antibodies in CRPS patients was 7.3%, showing characteristic ANNA and PCA patterns [25, 28]. A similar prevalence was found in healthy subjects, however, showing a clearly different, atypical pattern. The clinical relevance of such patterns is unclear but is observed more often in subjects without apparent neurological disease (Schreurs MWJ, unpublished results). Although the immunofluorescence pattern in the healthy population, reactivity to basket neurons and/or neurofilaments, was different as compared to the ANNA and PCA patterns observed in CRPS patients, this observation may lack meaning due to the low amount of positive patients identified.

The phenotype does not seem to be different, because the signs and symptoms did not show any significant differences between CRPS patients positive or negative for antinuclear antibodies, nor for antineuronal antibodies.

Our findings suggest that autoantibodies may be associated with the pathophysiology of CRPS, at least in a part of the patients. However, though being increased compared to the general population, the positive ANA prevalence in CRPS patients is much lower than in patients with classic systemic autoimmune disease such as systemic lupus erythematosus (SLE) that shows a prevalence of 99% [21]. The positive ANA prevalence in CRPS patients is more in line with the 25% observed in patients with autoimmune diseases such as rheumatoid arthritis (RA) [21]. Based on these findings, we may suggest that CRPS is more similar to an autoimmune disease as RA than to a systemic autoimmune disease as SLE. This hypothesis is supported by studies that revealed associations between CRPS and chronic inflammatory disorders, including asthma [10] and multiple sclerosis [11]. To our knowledge there are no reports of strong associations between CRPS and autoimmune diseases, although there are two case reports of cooccurrence of the two disorders in one patient [29, 30].

The presence of antineuronal antibodies in CRPS patients has been established in earlier research [15, 16]. In previous studies, antibodies were directed against a neuroblastoma cell line. In our current study we used a cerebellum substrate containing both afferent and efferent nerve pathways, with sensory and motor function. We chose this substrate because it resembles peripheral nerve tissue, which seems to be affected in CRPS [31]. Therefore, based on our observation of characteristic ANNA and PCA reactivity in some patients, our results suggest that autoimmunity against the peripheral nerve system could be of relevance in CRPS in a limited subset of cases. Since the majority of CRPS patients did not display antineuronal antibodies, a causal relationship remains to be determined. Alternatively, in the subset of patients with antineuronal antibodies, their expression might have been a secondary event as a result of nerve damage [32]. It would therefore be interesting to search for signs of actual nerve damage in patients who display these antineuronal antibodies and to search for the actual antigenic specificity of the antibodies. To define whether or not antineuronal antibodies could be causative for CRPS is of clinical relevance, as immune therapies, such as corticosteroids and intravenous immunoglobulin, have been shown to positively affect the neurological outcome when a disorder is caused by an antineuronal antibody directed against a cell surface antigen [22]. Interestingly, a previous work has already shown that some CRPS patients do respond to intravenous immunoglobulin treatment [18].

Before speaking of clear evidence of an autoimmune etiology, Witebsky's criteria for an autoimmune disease should be considered [33]. These criteria include (1) demonstration of a specific antigen, (2) circumstantial evidence of an autoimmune or inflammatory disorder from clinical clues, and (3) reproduction of clinical features in recipient animals by passive transfer of putatively pathogenic antibodies. We argue that CRPS definitely meets the second criterion. There are indications that the first criterion is met as well; however, this applies only to some of the CRPS patients. And more research is needed to define specific antigens involved. Injection of serum-IgG from a CRPS patient into groups of mice showed abnormal physical behavior and a significant reduction in rearing [34]. However, these findings are not a reproduction of the clinical features, as needed for the third criterion. Therefore, although suggestive, it remains uncertain whether CRPS can be defined as an autoimmune disease.

In conclusion, our findings indicate an autoimmune-related pathophysiology of CRPS in at least a subgroup of CRPS patients. Further research is needed into defining this subset and into the role of antibodies in the pathogenesis of CRPS in these patients.

## Figures and Tables

**Table 1:** Patients' characteristics.

Characteristics	*n* = 82
Woman (*n*, %)	69 (84.1)

Age in years (mean, SD)	44.2 (12.37)

CRPS duration in months (median, IQR)	11 (36–5)

Cold CRPS (*n*, %)	44 (53.7)
Warm CRPS (*n*, %)	31 (37.8)
Unknown (*n*, %)	7 (8.5)

Upper limb (*n*, %)	48 (58.5)

Precipitating injury	
Trauma (*n*, %)	53 (64.6)
Operation (*n*, %)	21 (25.6)
Spontaneous (*n*, %)	6 (7.3)

**Table 2:** IF pattern of antinuclear antibodies.

Antinuclear antibodies positive	*n* = 27
Homogeneous (*n*, %)	7 (26)
Speckled (*n*, %)	6 (22)
Nucleolar (*n*, %)	12 (44)
Homogeneous and nucleolar (*n*, %)	2 (8)

**Table 3:** IF pattern of antineuronal antibodies.

Antineuronal antibodies positive	*n* = 6
Neuronal nuclei (*n*, %)	4 (66)
Purkinje cells (*n*, %)	1 (17)
Neuronal nuclei and Purkinje cells (*n*, %)	1 (17)
